# Exploring the experiences related to postpartum changes: perspectives of mothers and healthcare providers in Iran

**DOI:** 10.1186/s12884-020-03504-8

**Published:** 2021-01-05

**Authors:** Mahboobeh Asadi, Mahnaz Noroozi, Mousa Alavi

**Affiliations:** 1 Student Research Committee, School of Nursing and Midwifery, Isfahan University of Medical Sciences, Isfahan, Iran; 2 Faculty of Nursing and Midwifery, Department of Midwifery, Isfahan (Khorasgan) Branch, Islamic Azad University, Isfahan, Iran; 3 Department of Midwifery and Reproductive Health, School of Nursing and Midwifery, Isfahan University of Medical Sciences, Isfahan, Iran; 4 Department of Psychiatric Nursing, School of Nursing and Midwifery, Isfahan University of Medical Sciences, Isfahan, Iran

**Keywords:** Maternal, Postpartum, Experience, Qualitative study, Iran

## Abstract

**Background:**

Numerous changes occur in different aspects of women’s lives in the postpartum period. Women’s adjusting with problems and taking advantage of this opportunity can develop their personality. In this regard, accurate knowledge of their experiences and feelings is necessary to help them to benefit from this period. Therefore, the present study aimed to explore the experiences related to postpartum changes in women.

**Methods:**

In the present qualitative study, 23 participants, including women of childbearing age who gave birth and healthcare providers (midwives and obstetricians) in Isfahan, Iran were selected using purposive sampling with a maximum variation strategy. Data were collected through in-depth semi structured interviews, field notes, and daily notes, and simultaneously analyzed using the conventional qualitative content analysis.

**Results:**

The data analysis results led to the extraction of three main categories including “feeling of decreased female attractiveness” (with two sub-categories of “ feeling of decreased beauty” and “feeling of decreased sexual function”), “feeling of insolvency and helplessness” (with two sub-categories of “physical burnout”, and “mental preoccupations”) and “beginning a new period in life” (with three sub-categories of “changing the meaning of life”, “feeling of maturity” and “deepening the communication”).

**Conclusions:**

Findings of this study can provide a good context for designing interventions to improve the women’s quality of life by explaining and highlighting their experiences in the postpartum period. In this regard, providing sufficient empathy, social and psychological support from family members (especially husband), performing appropriate educational interventions and also regular assessment of women’s psychological state by healthcare providers in postpartum period can reduce their concerns and help to improve their health.

**Supplementary Information:**

The online version contains supplementary material available at 10.1186/s12884-020-03504-8.

## Background

As a neonate enters the new world, the mother and other family members also enter a new world in such a way that extensive changes are necessary in their lifestyles, roles, and functions to maintain the physical, psychological, and social balance of family members [1]. These changes are more evident for mothers, so that a woman, who was in stable physical condition and had certain responsibilities in the housework, job, and social duties, will need care after her birth due to physical problems such as fatigue, episiotomy pain, caesarean section (CS) incision pain, and breast problems [2]. Furthermore, she must take care of a neonate who affects her communication [3]. Under such circumstances and due to the extent of changes, mothers experience a combination of different feelings, including love, happiness, stress, worry, and even shock [3, 4]. Sometimes the mothers’ stress and tension is so great that lead to her psychological problems in this period. The high prevalence of these problems highlights the importance of the issue, as postpartum depression occurs in 10 to 20% of women [5]. According to studies, the health of other family members in the postpartum period also depends on the mother’s health. Escribà-Agüir and Artazcoz found that the occurrence of postpartum depression in women could predict depression and anxiety in their husbands [6]. It has also been found that postpartum psychological problems have heavy economic costs for the healthcare system. A UK study estimated that the pregnancy-related depression, anxiety, and psychiatric disorders impose about 8.1 billion pounds of annual cost on society [7]. However, the postpartum period can become an enjoyable experience in women’s live; and facing challenges will provide an opportunity to increase their skills and personal growth [2]. De Caroli and Sagone determined the postpartum experience of support and stress. According to their results, the level of perceived postpartum stress was higher than before delivery and the level of perceived postpartum support was lower [8].

Matvienko-Sikar et al. investigated the factors affecting the stress of parents with 9-month-old children and indicated that mothers’ stress levels were higher than fathers; and stress in mothers was related to factors such as attachment, maternal health status, average sleep time, job and family income, while fathers’ stress was related to their attachment and health status [9]. Therefore, due to the fact that the postpartum period is a challenging period when mothers need help, it is necessary to have a comprehensive understanding of their feelings and experiences. Recognizing the experiences of women in the postpartum period can be effective in providing the necessary interventions to solve their problems and ultimately improve their health. Given that the qualitative research is an approach to discover and describe the participants’ experiences, it can increase insight and awareness of human experiences [10]. Therefore, the present study aimed to explore the experiences related to postpartum changes in women.

## Methods

### Study design

The present study was a part of an expanded mixed methods study which conducted from August 2019 to January 2020 and used a content analysis approach.

### Settings, sample and recruitment

In the present study, the participants were 17 women who gave birth and six healthcare providers (three midwives and three obstetricians) in Isfahan, Iran. These women were selected using the purposive sampling with a maximum variation strategy in terms of age, education, occupation, number of delivery, and type of delivery. Also, the midwives and obstetricians were selected using the purposive sampling with a maximum variation strategy in terms of work experience duration. Inclusion criteria included the participants’ willingness to participate in the study, informed consent, ability to express experience, Iranian citizenship, 2–24 months after the birth of a live and healthy neonate, and the absence of known major psychological disorders and chronic diseases. Access to participants was through health centers, hospitals, and private offices of obstetricians and midwives. After reaching eligible participants, none of them refused to participate in the study. They were recruited face to face or by phone calls. The first author (MA) had no previous contact or relationship with participants and centers. Table 1 presents the participants’ demographic characteristics.

**Table 1:** Demographic characteristics of participants

**Postpartum** **Women** **(*n*** **= 17)**	**Age (years)**	19–41
	**Level of education**	High school (2), High school diploma (5), Associate’s degree and B.S. (7), M.S. and Ph.D. (3)
	**Job status**	Employed (6), Housewife (11)
	**Type of employment**	Full time (5), Part-time (0), Casual (1)
	**Economic status**	Low (5), Middle (8), High (4)
	**Pregnancy number**	1–3
	**Number of delivery**	1–3
	**Number of children**	One child (9), Two children (6), three children (2)
	**Type of delivery**	Vaginal (11), Caesarean section (6)
**Healthcare providers (*n*** **= 6)**	**Occupation**	Midwife (3), Obstetrician (3)
	**Age (years)**	35–65
	**Working experience (years)**	7–35

### Data collection

Data collection methods included the semi-structured in-depth interviews, field notes, and daily notes. The first author (MA) conducted the interviews and field notes. She had 7 years working experience in midwifery and was Ph.D. candidate in reproductive health. Other authors have previous interviewing experience and qualitative paper/report writing. Interviews with postpartum women began with a general question, “Please explain your experiences about the motherhood and postpartum changes?” followed by open and interpretive responses by participants. Interviews with healthcare providers began with a general question “What do women experience in the postpartum period while taking the maternal responsibility? Please explain about it?” And then their open and interpretive answers guided the interview process (See additional files 1, 2 for copies of the topic guides). The time and places of the interviews determined based on the participants’ desire. Duration of interview sessions lasted from 30 to 90 min and was recorded by a digital tape recorder. The interviews continued until the data saturation occurred; when no new data was added to the existing data. In the present study, the first author (MA) recorded the observation of participants’ nonverbal behaviors, attitudes, and interactions in field notes during the interviews. The first author also asked the women who gave birth to record daily issues, as they wished, about their postpartum experience and feelings (daily notes) and deliver it to the her.

### Data analysis

Data analysis was manually performed and no software was used for this purpose. After completing the first interview, the data analysis performed using the conventional content analysis method. The interviews were transcribed verbatim by the first author (MA). The interviews were then reviewed repeatedly to gain a complete understanding of them. The sentences and phrases were then inductively coded, and those with a similar meaning were grouped together to create sub-categories. Thereafter, by comparing the sub-categories with each other, those which were conceptually related to each other were placed in a main category.

### Rigor and trustworthiness

To confirm credibility of the data, in other sessions, the coded interviews shared with three participating postpartum women, and summarized their final opinions (member checking). Furthermore, other methods utilized such as covering a wide range of participating postpartum women (in terms of age, education, occupation, number of delivery, and type of delivery) and combining different methods of data collection such as in-depth interviews, field notes, and daily notes. In the present study, two other authors who were knowledgeable in qualitative data analysis, assisted the first author to confirm the data. First, they rechecked the coded scripts, and mentioned their opinions about the coding method; later they suggested their own code lists. Regarding dependability of the data, the opinions of three experts used to match and ensure the consistency of findings with the participants’ statements (external checking). In order to increase transferability, the findings were presented to three postpartum women with similar characteristics of participating women who did not participate in the study to judge the similarity of the research results with their experience.

### Ethical approval

The research was approved by the Ethics Committee of the Research Deputy of Isfahan University of Medical Sciences (approval code: IR.MUI.RESEARCH.REC.1397.476); and informed consent, anonymity, confidentiality of information and the right to withdraw at any time were observed. Also, the reasons for the study were explained prior to each individual interview.

## Results

After the data analysis, 72 codes, seven sub-categories, and three main categories were obtained. Three main categories include: “feeling of decreased female attractiveness”, “feeling of insolvency and helplessness” and “beginning a new period in life” (Table 2).

**Table 2:** Results of the data analysis

Code	Sub -category	Main category
Feeling of loss of fitness	Feeling of decreased beauty	Feeling of decreased female attractiveness
Feeling of decreased physical attractiveness		
Feeling of less sexual attractiveness	Feeling of decreased sexual function	
Feeling of less sexual desire		
Physical problems	Physical burnout	Feeling of insolvency and helplessness
Heavy burden of duties and responsibilities		
Concerns about baby health	Mental preoccupations	
Concerns about returning physical health to pre-pregnancy condition		
Evaluate parental performance		
Child, the most important motivation to continue living	Changing the meaning of life	Beginning a new period in life
Child, the first priority of life		
Feeling of empowered	Feeling of maturity	
Higher resilience		
Feeling of perfection		
Changing the relationship with husband	Deepening the communication	
Changing the relationship with family members		
Changing the relationships with friends and relatives		

### Feeling of decreased female attractiveness

The postpartum women’s narrations indicated a decrease in their female attractiveness so that they experienced a decrease in beauty and sexual function by comparing their postnatal status with the pre-pregnancy period. This main category includes two sub- categories.

#### Feeling of decreased beauty

The participating postpartum women considered the breast enlargement and reshaping, overweight, loose abdominal skin, striae gravidarum on the abdomen and thighs, darkening of the skin, and melasma unpleasant, and expressed dissatisfaction with them. They narrated that observing such changes in their appearance and comparing themselves with the situation before pregnancy caused them to feel decreased fitness and beauty. A postpartum woman with a high school education who was a housewife said:

*“… Being overweight is very annoying for me! I do not like to buy new clothes and I think no clothes fit me!!!” (Second delivery)*

A postpartum woman with a master’s degree who was a university instructor said:

*“… The blemishes on my face and my skin looseness are so ugly!!! I feel bad comparing myself to the pre-pregnancy photos and I want to get back to my former beauty as soon as possible.”* (*First delivery)*

#### Feeling of decreased sexual function

The participating postpartum women narrated that they felt that they were not as sexually attractive as the past (due to the vaginal loosening) and not being well-groomed. A number of women also complained of scars at the episiotomy site and protrusion of labia minora in their external genitalia, citing a decrease in sexual attractiveness and subsequent decreased self-confidence. However, a number of postpartum participating women narrated that they had less anxiety and better sexual performance when they were approved by their husbands despite changes in their appearance. A postpartum woman with an associate’s degree who was a housewife said:

“… *I think I have lost my attractiveness! My vagina is loose, and I feel that sex is unpleasant for my husband... My husband tries not to touch my breasts during sex because he feels bad when he sees my breast milk! His reaction and sensitivity lowers my self-confidence.”* (*Second delivery)*

The participating postpartum women also narrated that they did not enjoy sexual intercourse due to the sense of burning and dryness, as well as dyspareunia, and felt that they had lower sexual desire in the postpartum period. A postpartum woman with a Ph.D. degree who was a university assistant professor said:

*“… My libido is not the same as before and I feel a lot of dryness and pain in my vagina during the intercourse. Sex has become very difficult for me!!!”* (*First delivery)*

### Feeling of insolvency and helplessness

According to the participants, they felt that they had no control over their situation and could not manage it due to the physical burnout and mental preoccupations in the postpartum period.

#### Physical burnout

The participants’ experiences indicated that physical burnout was due to the problems in the postpartum period and the burden of their duties and responsibilities. Participating women reported the experience of recurrent nocturnal awakenings, breast pain and discomforts, digestive problems (e.g. constipation, bloating, and hemorrhoids), headaches, and feelings of weakness and fatigue in the postpartum period. A postpartum woman with a high school diploma education who was a housewife said:

“… *My physical strength has decreased and I feel a sense of weakness at most of the day.*” (*First delivery*)

According to participating healthcare providers, postpartum women have the previous roles and responsibilities and are also responsible for continuous child care and frequent breastfeeding (especially in the first 6 months after delivery). It takes a lot of time and deprives them of the opportunity to get enough sleep and rest, and meet their personal needs. One of the midwives with 18 years of working experience said:

“*Some of my clients have to perform various responsibilities at the same time after giving birth. This tasks makes them unable to rest and take care of themselves, and even in some cases, this fatigue and exhaustion is completely recognizable from their appearance! … They often complain of feeling extremely tired.*”

#### Mental preoccupations

The participants’ experiences reflected the various mental preoccupations of women in the postpartum period so that they expressed concerns about the health, nutrition, and proper growth of their child, as well as problems such as jaundice and eczema. A postpartum woman with a bachelor’s degree who was a nurse said:

“… *I am very worried that something will happen to my baby!!! I wake up at night and check if my baby is breathing or not?”* (*First delivery*)

Participating healthcare providers believed that women, who had complications during pregnancy including gestational diabetes, pregnancy induced hypertension etc. were more likely to be concern about returning to the pre-pregnancy physical health. One of the obstetricians with 15 years of working experience said:

*“… in cases in which postpartum women have complications such as gestational diabetes or hypertension, they are worried about the complete return of their health.”*

Also, a number of participating postpartum women evaluated their parental performance by constant comparison of their behaviors with other mothers. A postpartum woman with a bachelor’s degree who was a housewife said:

*“… I am very worried about my child’s nutrition and I care less about myself, but my friend takes great care of herself and believes that her child needs a healthy and strong mother. I do not know which one of us are behaving properly?”* (*Second delivery*)

### Beginning a new period in life

The participants’ experiences indicated a change in the meaning of life after the birth, a sense of maturity, and deepening the communication in various aspects of life, and in other words, beginning a new period in their lives.

#### Changing the meaning of life

The participating postpartum women considered the birth of a child as a wonderful event and expressed that the child was as a motivation to continue their life. They described motherhood as the most beautiful feeling; and watching the child growth as the most enjoyable part of their lives. A postpartum woman with a bachelor’s degree who was a teacher said:

*“… Motherhood is truly the most beautiful feeling that God has created! I love life more since I have become a mother, and my motivation to continue living has increased.”* (*First delivery*)

Most of the participating postpartum women narrated that the child takes the most important place in the mother’s heart and becomes the first priority of her life so that all plans and daily affairs of her life are directed towards the growth and upbringing of the child. A postpartum woman with a master’s degree who was a university instructor said:

*“… I have changed my work schedule in a way that my child will have the least harm, even if it is difficult for me. For example, I have reduced my entertainment to take better care of my child because my child is the most important basis of my life!”* (*Second delivery*)

#### Feeling of maturity

A number of participating postpartum women described their experience of motherhood as a kind of “attainment of perfection” and narrated that they felt fulfilled with their child. A postpartum woman with a bachelor’s degree who was a housewife said:

*“…. After becoming a mother, I feel that the philosophy of my creation was to give birth to my child.... I feel so good*”!!! (*First delivery*)

A number of participating postpartum women also stated that since becoming mothers, they have felt less dependent on others and empowered in many affairs. A number of participating women also narrated that they have become more patient, and felt more resilient while facing difficult situations. A postpartum woman with a bachelor’s degree who was a housewife said:

*“… The big change after I become a mother is my patient. In the early of postpartum period, my baby had eczema, I was so stressed that visited several doctors within 2 to 3 days, but now (after 5 months), I feel patient!”* (*First delivery*)

#### Deepening the communication

The participating postpartum women believed that women’s postpartum communication changed in different dimensions so that with the birth of a child, the woman’s communication network is reorganized. They narrated that in the postpartum period, the relationship with the spouse changes and takes on a new status in the form of cooperation to care for the child. In some cases, the couple even tries to solve their old communication problems to ensure and maintain the health and happiness of the child. However, a number of participating postpartum women narrated that their relationships with their husbands have weakened in the postpartum period and felt that they did not get enough attention by their husbands. A postpartum woman with a bachelor’s degree who was a bank employee said:

*“… After giving birth, the relationship with the spouse significantly changes! The child is at the center and the parents enter into a partnership to take care of the child.”* (*Third delivery*)

Participating postpartum women narrated that after giving birth and becoming mothers, they understood their parents more and felt more affection and belonging to them. Also, a number of participating postpartum women narrated that they strengthened their relationships with supportive relatives and friends, as well as their peers (with whom they could share their maternal experiences) in the postpartum period. A postpartum woman with a high school diploma education who was a housewife said:

*“After giving birth, I mostly communicate with friends who have children, and I try to communicate more with those who are good guides for the motherhood.”* (*Second delivery*)

## Discussion

The present study aimed to explore the experiences related to postpartum changes. In the study, the participants’ narrations indicated feeling a decrease in the female attractiveness so that postpartum women experienced a decrease in their beauty and sexual functions by comparing them with the pre-pregnancy situation. Schlagintweit et al. found that many women had negative body image due to many physical changes after the childbirth. In addition to worrying and dissatisfaction about losing their beauty, women also worry about decreasing their sexual attractiveness [11]. According to researchers, women’s libido decreases in the postpartum period due to the hormonal changes, vaginal dryness, dyspareunia, fatigue and waking up at night, and eventually they will have the impaired sexual function [2]. In such situations, education have a significant impact and greatly reduce concerns. Women should know that the return of changes in their appearance is a long process and their sexual function is affected by certain conditions after childbirth. Couples also need to be trained to interact properly during this period [2, 12]. According to results of the present study, women who were approved by their husbands, had less anxiety and better sexual performance despite their appearance changes. Results of other studies have indicated that the couples’ feeling of emotional closeness, empathy, and support of their spouses are important factors affecting the women’s sexual function in the postpartum period [13, 14].

In the present study, the participants’ experiences showed feelings of insolvency and helplessness in women in the postpartum period. Women in the postpartum period need a period of energy recovery and extensive physical changes that take about 8 weeks. In this situation, women are also responsible for caring for their babies in addition to having prenatal duties. Now, if they do not have enough support in this period, they will suffer from physical burnout [6, 15]. Ospina et al. also considered postpartum period as a period when women need the comprehensive assistance [2]. Also, Matvienko-Sikar et al. cited the lack of adequate support as a main cause of perceived postpartum stress [9]. Therefore, in the postpartum period, family members (especially the husband) should help the mother with an adequate social and psychological support. Also, regular assessment of women’s psychological state by healthcare providers in postpartum period are recommended.

In the present study, postpartum women had many mental preoccupations and the baby’s health, their functions as a good parent, and the return of physical health (in cases such as gestational diabetes and hypertension) were the main concerns of them. Figueiredo and Conde believed that due to many changes in the world of women after childbirth, the presence of stress was somewhat normal [16], but in some cases, the higher stress could cause anxiety and depression in women [6]. In these cases, support and reassurance are very helpful. When women are aware of the symptoms of health risks, their worries about the health of baby and themselves decrease [2]. In many cases, women like to learn from other mothers and talk to them about their experiences, performances and problems in the postpartum period. By doing so, they no longer feel alone, their expectations of themselves become more realistic, they feel better self-efficacy, and consider themselves as capable mothers [3, 17].

In the present study, the participants’ experiences indicated the feeling of beginning a new period in the women life in the postpartum period. Many participating women stated that a new motivation was formed in them to continue living after the birth of their child. They also narrated that they become more patient and self-sacrificing and are more resilient while facing the problems after giving birth. Consistent with these results, in a study by Shaikh and Kauppi, mothers also talked about discovering new strengths in themselves [17]. In a study by Piontkowski, the results also indicated that after the birth of a child, the mother sees her place different in the world [18].

In the present study, the participating women stated that their relationships with their husbands had taken a new form of cooperation after childbirth, and even in some cases, the previous communication problems with their husbands were consciously resolved in favor of raising their child. However, in a number of cases, women narrated that their relationships with their husbands have weakened. In a study by Entsieh & Hallström, women were also dissatisfied with changes in relationships with their husbands and considered it necessary to have information about how couples interacted after childbirth [3]. In this regard, the need to educate couples on how to interact with each other and meet their needs in the postpartum period was evident.

In the present study, the participating women narrated that feeling more intimate with their parents, changing their relationships with friends and peers, and establishing deep and purposeful relationships (to better perform maternal duties and responsibilities) in the postpartum period. In this regard, results of a study by Nayak and Shetty also indicated that the relationship between the woman and her family, especially her mother and grandmother, became closer after childbirth, and this relationship played an important role in women’s adjustment and good feeling of being a mother [19].

Postpartum women with known psychological disorders (postpartum depression etc.) were not included in the study, but the presence of unknown psychological disorders may have affected the obtained data which is a limitation for this study.

## Conclusion

The present study showed women’s experiences related to postpartum changes including feeling of decreased female attractiveness, feeling of insolvency and helplessness and beginning a new period in life. In this regard, providing sufficient empathy, social and psychological support from family members (especially husbands), reassuring, performing appropriate educational interventions, and also regular assessment of women’s psychological state by healthcare providers can help them adjust to postpartum changes and improve their quality of life and health.

## Supplementary Information

**Additional file 1.** Interview guide during the face-to-face interviews with postpartum women for the study conducted to explore the experiences related to postpartum changes from the perspective of postpartum women and healthcare providers in Isfahan Town, Iran, 2019–2020 (See Methods section for further description).

**Additional file 2.** Interview guide during the face-to-face interviews with healthcare providers (midwives and obstetricians) for the study conducted to explore the experiences related to postpartum changes from the perspective of postpartum women and healthcare providers in Isfahan Town, Iran, 2019–2020 (See Methods section for further description).

## Data Availability

The datasets generated and/or analysed during the current research are not publicly available as individual privacy could be compromised but are available from the corresponding author on reasonable request.

## Abbreviation

CS: Caesarean section
