# Supplementary material for: Exploring the experiences related to postpartum changes: perspectives of mothers and healthcare providers in Iran
Source: BMC Pregnancy Childbirth. 2021 Jan 5;21:7. doi: 10.1186/s12884-020-03504-8 (PMC7783980; doi:10.1186/s12884-020-03504-8)
Supplement: Supplementary file 1 — Additional file 1. Interview guide during the face-to-face interviews with postpartum women for the study conducted to explore the experiences related to postpartum changes from the perspective of postpartum women and healthcare providers in Isfahan Town, Iran, 2019–2020 (See Methods section for further description). [file 12884_2020_3504_MOESM1_ESM.docx]

**Additional file 1:** Interview guide during the face-to-face interviews with postpartum women for the study conducted to explore the experiences related to postpartum changes from the perspective of postpartum women and healthcare providers in Isfahan Town, Iran, 2019-2020 (See methods section for further description).

**Introduction:** *Aim, to create appropriate atmosphere*

- Name of the interviewer and affiliation
- Purpose of the study
- Consent to take part in the study
- Confidentiality, explain how the data will be used
- Interview will last approximately 30-90 minutes
- Audio recorded to ensure interviewer can fully engage in the interview

**Warm up questions:** *Aim\ make participants comfortable*

1. Please introduce yourself?

2. How old are you?

3. What is your education level?

4. What is your job?

5. How many deliveries have you had?

6. What type of deliveries have you had?

**Interview guide questions in individual interviews with postpartum women**

1. Please explain your experiences about the motherhood?

2. Please explain your experiences about the postpartum changes?

3. What have you encountered in the postpartum period? Please explain?

4. What would you like your spouse to do for you in the postpartum period? Please explain?
