# Supplementary material for: Exploring the experiences related to postpartum changes: perspectives of mothers and healthcare providers in Iran
Source: BMC Pregnancy Childbirth. 2021 Jan 5;21:7. doi: 10.1186/s12884-020-03504-8 (PMC7783980; doi:10.1186/s12884-020-03504-8)
Supplement: Supplementary file 2 — Additional file 2. Interview guide during the face-to-face interviews with healthcare providers (midwives and obstetricians) for the study conducted to explore the experiences related to postpartum changes from the perspective of postpartum women and healthcare providers in Isfahan Town, Iran, 2019–2020 (See Methods section for further description). [file 12884_2020_3504_MOESM2_ESM.docx]

**Additional file 2:** Interview guide during the face-to-face interviews with healthcare providers (midwives and obstetricians) for the study conducted to explore the experiences related to postpartum changes from the perspective of postpartum women and healthcare providers in Isfahan Town, Iran, 2019-2020 (See methods section for further description).

**Introduction:** *Aim, to create appropriate atmosphere*

- Name of the interviewer and affiliation
- Purpose of the study
- Consent to take part in the study
- Confidentiality, explain how the data will be used
- Interview will last approximately 30-90 minutes
- Audio recorded to ensure interviewer can fully engage in the interview

**Warm up questions:** *Aim\ make participants comfortable*

1. Please introduce yourself?

2. How old are you?

3. What is your education level?

4. What is your job?

5. What is your work experience?

**Interview guide questions in individual interviews with healthcare providers**

1. In your opinion, what do women experience in the postpartum period? Please explain about it?

2. In your opinion, what do women face when taking on maternal responsibilities? Please explain about it?

3. In your opinion, what problems do women face in the postpartum period?
